# Color Stability of Polymer-Based Composite CAD/CAM Blocks: A Systematic Review

**DOI:** 10.3390/polym15020464

**Published:** 2023-01-16

**Authors:** Gaetano Paolone, Mauro Mandurino, Francesca De Palma, Claudia Mazzitelli, Nicola Scotti, Lorenzo Breschi, Enrico Gherlone, Giuseppe Cantatore, Alessandro Vichi

**Affiliations:** 1 Dental School, IRCCS San Raffaele Hospital, Vita-Salute University, 20132 Milan, Italy; 2 Department of Biomedical and Neuromotor Sciences, DIBINEM, Alma Mater University of Bologna, Via S. Vitale 59, 40125 Bologna, Italy; 3 Department of Surgical Sciences, Dental School Lingotto, University of Turin, Via Nizza 230, 10126 Turin, Italy; 4 Dental Academy, University of Portsmouth, Portsmouth PO1 2QG, UK

**Keywords:** polymer-based, hybrid ceramic, cad/cam, resin nano-ceramic, resin ceramic, composite, color stability, staining, color change

## Abstract

Background: This systematic review aims to evaluate the color stability of resin composite CAD/CAM blocks (CCB) when submitted to staining solutions. Methods: A systematic search was performed on different databases (Embase, Medline, Scopus, Web of Science). Search terms were: ‘polymer infiltrated’, ‘polymer-based’, ‘resin nanoceramic’, ‘resin ceramic’, ‘hybrid composite’, ‘hybrid ceramic’, ‘composite ceramic’, ‘resin infiltrated’, ‘CAD-CAM’, ‘CAD/CAM’, ‘color stability’, ‘staining’, ‘staining susceptibility’, ‘color change’, ‘color difference’. Inclusion criteria: in vitro articles published in the English language until 18 September 2022 without initial time restriction evaluating the color stability of CCB when submitted to staining solutions. Exclusion criteria: studies investigating color change induced by smoke or whitening treatments; studies not including a clinical evaluation of the results using the thresholds for color perceptibility and acceptability. Risk of bias assessment using the QUIN tool. Findings: Out of the 378 initially retrieved articles, 19 were included in this review. They investigated 17 different CCBs and different artificial staining by liquid protocols, including coffee, red wine, tea, and cola. CCBs exceeded clinical acceptability thresholds for color shift in 18 out of 19 studies, with a significantly higher color stability than conventional hybrid resin-based composites (RBCs), and a significantly lower color stability than ceramic materials. The identified differences in CCBs in color stability can be attributed to the material’s composition, but also to the heterogeneity of staining procedures. Interpretation and clinical implication: Clinicians should be aware that, although to a lower degree when compared to RBCs used in direct or indirect procedures, CCBs undergo color changes to a higher degree in comparison to ceramic materials.

## 1. Introduction

In less than 40 years, CAD/CAM technology has experienced constant hardware and software improvements that have resulted in easier use and more reliable clinical performance [1,2,3]. Along with progress in technology, new materials have been developed for CAD/CAM restorations, such as glass ceramics, zirconia, and composites, allowing clinicians the choice of different mechanical [4,5] and optical [6] properties. Although the use of direct resin-based composite (RBC) restorations is largely diffused and able to provide reliable and esthetic results both for anterior [7,8] and posterior [9] direct restorations, some drawbacks have been reported, such as weak mechanical properties [10] and lack of color stability.

When compared with RBCs used in direct restorations, indirect ones are characterized by higher mechanical properties and color stability, mainly due to the higher degree of conversion that can be obtained with extra-oral curing. Today, indirect RBCs are extensively used as a reliable alternative to ceramic restorations to produce inlays or onlays [11].

CAD/CAM composite blocks [12] (CCB) (also referred as resin nano-ceramic [13,14,15,16,17,18,19], resin ceramic [20], resin-based composites blocks [21,22,23,24], nanohybrid restorative materials [25], nano-hybrid ceramic [26], hybrid composites [27], hybrid ceramic [28]) have recently gained popularity as they are considered to offer a higher quality compared to composite used in a conventional manual procedure in both direct and indirect restorations. This is mainly due to the standardized industrial production processes, for which high temperature and/or high-pressure polymerization are used. These are able to maximize polymer cross-linking [12] and, therefore, the material’s properties. Other advantages of CCBs are that they are more easily finalized than ceramics, because after milling no firing process is needed. Furthermore, they are easy to finish and polish, and repair [12].

Since the introduction of the first CCB (MZ100, 3M ESPE, St. Paul, MN, USA) in the early 2000s [29], several CCBs have been marketed. Due to the heterogeneity of the available CCBs, it is worthwhile analyzing their behavior to understand possible clinical drawbacks. One of the known limits of resin-based materials is the propensity to color change in the long term due to intrinsic and extrinsic factors [30]. Intrinsic factors are related to the material’s composition, such as the type of resin matrix, fillers, and polymerization initiators [31]. Extrinsic factors are related to the absorption of pigments coming from external sources, such as food or drinks. Some commonly used beverages like coffee, tea, coke, red wine, and juices can affect the color stability of composite resins [32].

With patients’ increasing esthetic demands, the color stability of restorative materials has become pivotal in determining the long-term clinical success and the longevity of a restoration. Objectives: a comprehensive systematic literature review was conducted to investigate the color stability of CCBs when submitted to artificial staining. 

## 2. Materials and Methods

This systematic review followed the Preferred Reporting Items for Systematic reviews and Meta-Analyses (PRISMA) statement [33].

### 2.1. Eligibility Criteria

Population: We included in vitro studies analyzing color change of composite CAD/CAM blocks obtained by artificial staining by liquids. Examples of liquids we included: coffee, tea, cola, juices.

Outcome: To be included, articles should use a color-change formula to evaluate color stability.

Inclusion Criteria:

1. In vitro studies investigating color stability of composite CAD/CAM blocks;
2. In vitro studies including artificial staining procedures by liquids;
3. Studies using color difference clinical thresholds to analyze the color difference values;
4. Publications in English language;

Exclusion Criteria

5. In vitro studies with a sample size of less than five test specimens in each subgroup;
6. In vitro studies investigating color stability of hybrid dental ceramic CAD/CAM blocks (polymer-infiltrated ceramic networks);
7. In vitro studies investigating color stability of CAD/CAM materials for temporary restorations;
8. Clinical trials, case reports, reviews, or animal studies;
9. Papers analyzing color stability only with water aging/thermocycling procedures;
10. Papers analyzing color stability with whitening procedures;
11. Papers analyzing color stability with mouth rinses;
12. Papers analyzing color stability with smoking procedures;

### 2.2. Information Sources

One reviewer (GP) conducted a search for English language articles published in dental journals until 18 September 2022 in the following electronic databases: PubMed, Embase, Web of Science, Scopus. A manual search was also conducted.

### 2.3. Search Strategy

Searches used a combination of MeSH terms and free text words, as follows: ‘polymer infiltrated’, ‘polymer-based’, ‘resin nano-ceramic’, ‘resin ceramic’ ‘hybrid composite’, ‘hybrid ceramic’, ‘composite ceramic’, ‘resin infiltrated’, ‘composite’, ‘nano-hybrid’, ‘CAD-CAM’, ‘CAD/CAM’, ‘color stability’, ‘staining’, ‘staining susceptibility’, ‘color change’, ‘color difference’. All strategies were based on the search strategy developed for PubMed (Table 1) and were appropriately revised for each database to account for differences in controlled vocabulary and syntax rules.

### 2.4. Selection Process

For the selection of studies, two authors (G.P. and M.M.) independently reviewed titles and abstracts of the studies according to the inclusion criteria. Final inclusion of studies was based on screening and assessing full texts, and with consensus of the authors of the current review.

### 2.5. Data Items

An extraction form was used to collect retrieved data items: type of CCB, comparison with other materials, staining liquids, staining protocol, time of color assessment, type of spectrophotometer, color difference formula, specimens finished, specimen repolished, clinical thresholds, outcomes.

### 2.6. Study Risk of Bias Assessment

The risk of bias assessment used the QUIN tool (risk-of-bias tool for assessing in vitro studies conducted in dentistry) [34]. The study’s quality assessment was conducted according to a fixed set of domains of bias (Clearly stated aims/objectives; Detailed explanation of sample size calculation; Detailed explanation of sampling technique; Details of comparison group; Detailed explanation of methodology; Operator details; Randomization; Method of measurement of outcome; Outcome assessor details; Blinding Statistical analysis; Presentation of results). QUIN final assessment was performed by categorizing each of the study features at ‘low’, ‘medium’, or ‘high’ risk of bias. Both reviewers (G.P. and F.D.P.) independently conducted the assessment, and any uncertainties or disagreements were then resolved by discussion. 

## 3. Results

### 3.1. Study Selection and Study Characteristics

**Figure 1:**
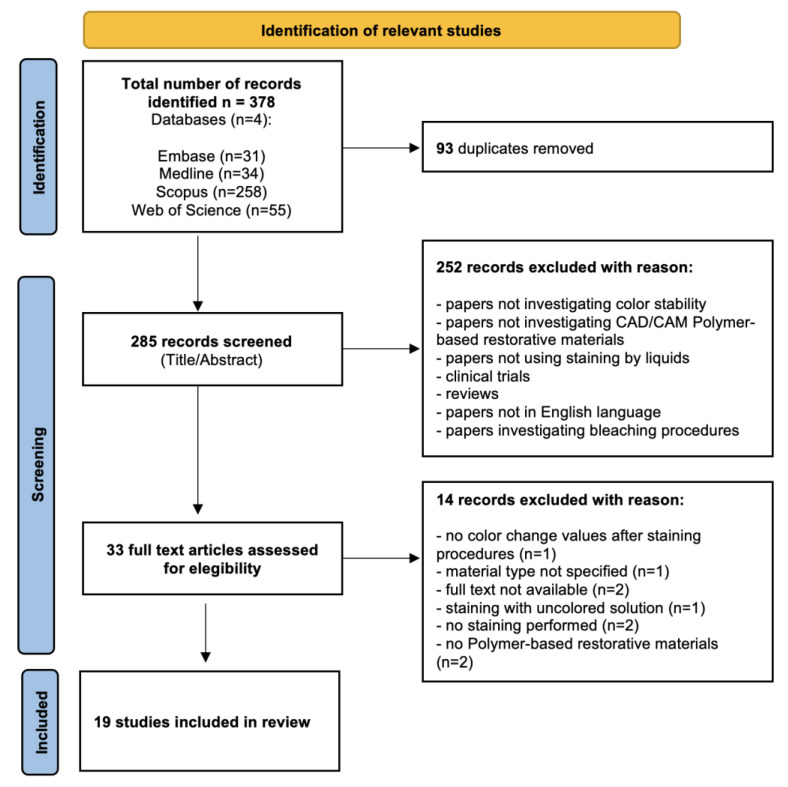
Identification of relevant studies.

The study selection process according to the PRISMA checklist is reported in Figure 1.

A total of 378 studies were identified through the initial database search. Following duplicates removal, 285 records were screened by title and abstract. During the screening process, 252 records were excluded as not relevant to the subject, and 33 were selected for full-text assessment. Finally, 19 studies were included in this systematic review as they met the inclusion criteria. 

### 3.2. Composite Block Specimen Characteristics

Among the identified studies, 17 (n = 17) composite blocks were investigated. Composition of these materials is listed in Table 2. The most studied CCB (n = 12) was Lava Ultimate (3M Espe, St. Paul, MN, USA), followed by Cerasmart (GC, Bunkyo-ku, Tokyo, Japan) (n = 5).

Data from the retrieved papers were chronologically reported in two predefined data extraction forms (Table 3 and Table 4).

### 3.3. Artificial Staining Procedures

Artificial staining procedures were different among the retrieved studies. The staining solutions used were coffee (n = 15), red wine (n = 9), cola (n = 7), tea (n = 5), ginger (n = 2), and juice (n = 2). Specimens were immersed in the staining media for different time periods that ranged from 2 days to 12 weeks. Most of the studies renewed liquids at different intervals. The immersion was static and ran from some minutes per day to continuous immersion for the entire staining periods. Most of the studies kept the liquid temperature stable at 37°C (n = 13). For other studies, the liquids were kept at room temperature (n = 2) or thermocycled (n = 2). In two studies, information on storage temperature was not reported. 

### 3.4. Color Assessment

Seventeen studies out of the nineteen examined used a spectrophotometer to assess color change, while two used a spectroradiometer. Among the spectrophotometers, the most used (n = 8) was EasyShade (Vita Zahnfabrik, Bad Säckingen, Germany). In ten papers, the CIEDE2000 color difference formula was used, while in seven the CIELAB formula was used. In only two studies were both formulas used. Seventeen papers compared the color stability of CCB with other materials, while two compared different CCBs.

### 3.5. Surface Treatment

Sixteen of the retrieved studies performed surface finishing and polishing before staining procedures. These procedures were fairly uniform among the studies because fourteen out of sixteen used silicon carbide abrasive papers (and twelve with 1200 grit as a final step) and two used abrasive disks. Only one paper performed a repolishing step after the staining procedure.

### 3.6. Clinical Thresholds and Comparison with Other Materials

CCBs showed color change beyond clinically acceptable thresholds in all studies except for one [15]. When compared with other materials, CCBs immersed in coffee solutions showed a significantly higher color change than lithium disilicate [13,15,19,28,35,36,37], zirconia-reinforced lithium silicate [15,22], hybrid ceramic [17,18,19,21,35,36,37], and feldspatic ceramic [13,26,37]. CCBs immersed in red wine showed a significantly higher color change than zirconia-reinforced lithium silicate [13,19,22,38], hybrid ceramic [14], and feldspatic ceramic [13,24]. Conversely, CCBs showed higher color stability when compared with hybrid resin composites for direct restorations [12,28].

### 3.7. Qualitative Assessment of the Investigations

The Quality Assessment Tool For In Vitro Studies (QUIN Tool) was used to evaluate in vitro papers included in this review (Table 5). A high risk of bias could be found in all RCTs except for one [36] regarding blinding, mostly owing to the fact that blinding of participants and personnel was not applied or declared. Randomization was clearly stated in only five investigations [15,19,27,28,36]. Eleven studies reported complete information regarding sample size calculations [12,14,16,19,24,25,26,28,36,37,38], eight regarding sampling technique [12,15,16,25,27,28,37,38], and two regarding the comparison (control) group [28,36].

## 4. Discussion

In recent years, the request for esthetic dental restorations has considerably increased. Today, RBCs are the most widely used materials for direct and for indirect restorative procedures because of their excellent esthetic and mechanical properties [39,40].

CCBs are claimed to provide better mechanical and optical properties than their traditional direct and indirect resin counterparts thanks to the benefits of the industrial production processes [12]. Amid other advantages, they are claimed to reduce one of the primary drawbacks of direct and indirect RBCs, which is color stability. This can compromise the esthetic outcomes of the restorations over time [41]. Despite their increasing use, very little is known on the color changes of CCBs. Therefore, the purpose of this review was to evaluate the color stability of CCBs.

### 4.1. Type of Material

Regarding the examined materials, Lava Ultimate was the most investigated, followed by Cerasmart. Acar et al. [35] reported that Lava Ultimate, after 5,000 thermocycles in coffee, showed color change values higher than the clinical acceptability threshold when compared to lithium disilicate and polymer-infiltrated ceramic network (Enamic). This result, despite the paper presenting a high risk of bias, could be related to the composition of the material: Lava Ultimate consists of a hydrophobic urethane-dimethacrylate (UDMA) and a hydrophilic triethylene-glycol-dimethacrylate (TEGDMA). TEGDMA is generally added to the composition of RBCs because it is more viscous than bisphenol-glycidyl-methacrylate (Bis-GMA) and permits copolymerization, diluting Bis-GMA and increasing composite sculptability. TEGDMA, however, increases the hydrophilicity of the composite, resulting in an increased susceptibility to staining [42,43].

Lava Ultimate contains Bis-GMA and its ethoxylated form (Bis-EMA). Dental materials containing Bis-GMA show the highest degree of water sorption and, therefore, liquid dyes, when compared with those based on UDMA, TEGDMA, and BisEMA [44].

Al Amri et al. [36], Eldwakhly et al. [15], and Schürmann and Olms [17] confirmed Lava Ultimate’s lower color stability when compared to ceramic materials. Furthermore, these papers show low or medium risk of bias; therefore, their findings could be considered reliable.

Three papers included in this review compared Lava Ultimate with Cerasmart. Two of them showed significant higher color stability for Cerasmart [14,19], while the third, with a lower risk of bias, reported no significant differences [36]. The higher Cerasmart color stability may be related to the absence of Bis-GMA in its composition, thus confirming that this monomer is responsible for water uptake and, therefore, possibly for discoloration.

### 4.2. Spectrophotometric Analysis and Clinical Thresholds

Spectrophotometric analysis allows for an objective color comparison. Color coordinates are measured, and differences are compared by CIELAB or CIEDE2000 formulas, which are the most frequently used to analyze color changes [45]. The Perceptibility Threshold (PT) refers to the magnitude of color difference that is visually detectable by the human eye, while the Acceptability Threshold (AT) corresponds to the magnitude of color difference that is considered clinically not acceptable [46]. The CIEDE2000 color difference formula [47] is considered to be a better indicator of human capability to detect perceptible and acceptable color differences, and was used by the majority of the papers (10 out of 19) included in this review. Differences detected by spectrophotometers can be evaluated from a statistical point of view or by utilizing the PT or AT. The statistical outcome of color measurements should always be integrated with PT and AT to validate clinical consistency. For this reason, papers that did not take into consideration clinical thresholds [46] for the interpretation of the results were not included in the current review. The most frequently used instrument used for color measurements was the spectrophotometer. Among the spectrophotometers, Easyshade (Vita Zahnfabrik) was the most widely used. This type of spectrophotometer is designed to be a clinical device (working in “tooth mode”) and it is not recommended for in vitro testing. The results of studies performed with such a device should be cautiously evaluated, and a bench-top spectrophotometer should be preferred [48].

### 4.3. Staining Solution

The level of CCB color change, as with conventional RBCs, is closely related to the type of staining solution [49,50]. Depending on the staining liquid used to evaluate color stability, colorants can deposit either on the surface [51] or in the structure [52] of the tested material, or the liquid can induce staining, altering the surface because of low pH [49,50]. Probably due to its increased use among the population, coffee is the most investigated staining media in the papers evaluated in this review. Coffee induces staining through a yellow coloring pigment that is characterized by different polarities [30,53]. Red wine, the second most used solution in this review, has staining capability and contains alcohol that may lead to rough surfaces and, consequently, pigment adsorption [49,50,54]. It has also been reported that ethanol, contained in red wine, has a solvent effect on the monomers, increasing potential discoloration [55,56]. Other dyes investigated in the paper analyzed in the current review, such as tea, cola, energy drink, and juices, are responsible for CCB color changes, but to a lower degree compared to coffee and wine, generally below AT.

### 4.4. Effects of Surface Treatment on Discoloration

Some of the included studies investigated the effects of surface treatments on color stability of CCBs submitted to staining with colored dyes. Unlike conventional hybrid composites used in direct procedures, CCBs are not characterized by a surface-resin-rich layer, which could be responsible for higher color changes if not removed by finishing and polishing procedures [41]. However, it has been reported that after milling, finishing and polishing CCBs may reduce surface roughness and, therefore, staining [57]. Aydin et al. [23] reported that polishing a specimen’s surface produced a lower color change when compared with a control group (unpolished). However, both groups exceeded the AT, confirming that clinical thresholds should always be referenced for a correct data interpretation. Sagsoz et al. [20] also confirmed that polishing is crucial for all materials, as differences were observed between polished and unpolished specimens. Moreover, the authors reported significant differences in color stability when different finishing and polishing systems were used, suggesting that each material requires a specific finishing/polishing system for optimal performance.

## 5. Conclusions

Based on the findings of this systematic review, and considering the risk of bias, the following conclusion can be drawn:

1. Resin-based blocks for CAD/CAM procedures show higher color stability than direct or indirect (laboratory) RBCs;
2. Resin-based blocks for CAD/CAM procedures show lower color stability than ceramic materials;
3. The color stability of CCBs mainly depends on material composition and staining media, but finishing/polishing procedures also have an influence.

## Figures and Tables

**Table 1:** Search conducted in Medline/PubMed database.

Search	Query
#1	“color difference* “[All Fields] OR “color change” [All Fields] OR “color stability” [All Fields] OR “colour difference * ”[All Fields] OR “colour change” [All Fields] OR “colour stability” [All Fields] OR “staining” [All Fields] OR “stain susceptibility” [All Fields]
#2	“polymer infiltrated” [All Fields] OR “polymer-based” [All Fields] OR “resin nanoceramic * ” [All Fields] OR “resin ceramic *” [All Fields] OR “hybrid composite *” [All Fields] OR “composite ceramic * ”[All Fields] OR “hybrid ceramic * ”[All Fields] OR “resin infiltrated” [All Fields]
#3	“computer aided design” [MeSH Terms] OR (“computer aided” [All Fields] AND “design” [All Fields]) OR “computer aided design” [All Fields] OR (“cad” [All Fields] AND “cam” [All Fields]) OR “cad cam” [All Fields]
#4	#1 AND #2 AND #3

**Table 2:** Composition of CCBs investigated by papers included in current review.

Product	MATRIX	FILLER	Manufacturer
Lava Ultimate	BisGMA, UDMA, BisEMA, TEGDMA (20 wt%)	SiO_2_ (20 nm), ZrO_2_ (4–11 nm), aggregated ZrO_2_/SiO_2_ microcluster (80 wt%)	3M ESPE, St. Paul, MN, USA
Paradigm MZ 100	Bis-GMA, TEGDMA (15 wt%)	ultrafine zirconia-silica ceramic (85 wt%)	3M ESPE, St. Paul, MN, USA
Brilliant Crios	Cross-linked methacrylates (Bis-GMA, Bis-EMA, TEGDMA) (30 wt%)	Glass and amorphous silica (30 wt%)	Coltene, Switzerland
Crystal Ultra	Cross-linked polymer (BisGMA, UDMA, BUDMA) (30 wt%)	Ceramic-like inorganic silicate glass fillers (70 wt%)	Digital Dental, Scottsdale, AZ, USA
Brava Block	Methacrylate monomers	initiator, co-initiator, stabilizers, silane, glass-ceramic particles, silica, and pigments.	FGM Dental Group
Cerasmart	BisMEPP†, UDMA, DMA (29 wt%)	Silica and barium glass nanoparticles (Silica (20 nm), barium glass (300 nm)) (71 wt%)	GC America, Alsip, IL, USA
Cerasmart 300	BisMEPP†, UDMA (22 wt%)	Silica and barium glass nanoparticles (Silica (20 nm), barium glass (300 nm)) (78 wt%)	GC America, Alsip, IL, USA
Katana Avencia Block	UDMA, TEGDMA	silica, alumina filler	Kuraray, Japan
Katana Avencia P Block	UDMA	Ba-glass, silica	Kuraray, Japan
Shofu HC Block	UDMA + TEGDMA (39 wt%)	Silica-based glass and silica (61 wt%)	Shofu, Japan
Estelite Block	UDMA, TEGDMA (25 wt%)	Silica, silica-zirconia (75 wt%)	Tokuyama Dental, Japan
Estelite P Block	Bis-MPEPP, UDMA, NPGDMA (19 wt%)	Silica, silica-zirconia (81 wt%)	Tokuyama Dental, Japan
Duro Ace	UDMA, Bis-EMA (15 wt%)	Silica, Ba-glass (85 wt%)	Vericom, Chuncheon, Korea
Mazic Duro	UDMA + TEGGDMA (23 wt%)	Barium aluminosilicate, silicon dioxide and zirconia (77 wt%)	Vericom, Chuncheon, Korea
Grandio Blocs	UDMA + DMA (14 wt%)	Nanohybrid filler (86 wt%)	VOCO GmbH, Germany
KZR-CAD HR2	UDMA, TEGDMA (21 wt%)	SiO_2_ + Al_2_O_3_ + ZrO_2_, SiO_2_ (79 wt%)	Yamakin, Japan
KZR-CAD HR3	UDMA, DEGDMA (25 wt%)	SiO_2_ + Al_2_O_3_ + ZrO_2_, SiO_2_ (75 wt%)	Yamakin, Japan

**Table 3:** List of the in vitro studies included in the review after the screening process. CCB: CAD/CAM composite block; TC: thermocycling. (1: TC= thermocycling; 2: ∆E00= color change obtained with CIEDE2000 formula; 3: PICN= polymer infiltrated ceramic network; 4: ∆E= color change value obtained with CIELAB formula.).

First Author, Year	CCB	Comparison	Staining	Staining Protocol	Timeline	Spectrophotometer	Finishing/Polishing (Yes = y; No = n)	Repolishing	THRESHOLD (Perceptible = p; Acceptable = a)	Outcomes
Acar et al., 2016 [35]	Lava Ultimate (3 M, Seefeld, Germany)	Enamic; IPS e.max CAD; Filtek Supreme Ultra Universal	Coffee	5.000TC (5–55 °C, dwell time: 30 s, transfer time: 10 s) Renewal: 8 h	Baseline, coffee TC ^1^	Spectroradiometer	y	/	∆E00 ^2^ = 1.28 (p); ∆E00 = 2.24 (a)	Color change was beyond clinical acceptability for Lava Ultimate and Filtek Supreme Ultra Universal. The average color change of Vita Enamic was clinically perceivable over the tested thickness values. The color change of IPS e.max CAD was not clinically perceivable at any tested thickness
Al Amri et al., 2021 [36]	Lava Ultimate; Cerasmart; Crystal Ultra	IPS e.max-CAD; Vita Enamic	Coffee, distilled water (control)	5.000 TC (5–55 °C, dwell time: 30 s, transfer time: 10 s) Renewal: 1d	Baseline, T0 (5.000 TC), T1 (immersion in coffee or distilled water), T2 (further 5.000 TC)	Spectrophotometer (CM-2600d, Konica Minolta Sensing Inc., Osaka, Japan)	y	/	∆E00 = 0.8 (p); ∆E00 = 1.8 (a)	The Crystal Ultra exhibited better color stability compared to Lava Ultimate and Cerasmart, but had higher color change when compared with Vita Enamic PICN ^3^ and IPS e.max CAD.
Aydin et al., 2020 [23]	Cerasmart; Shofu Block; Grandio Blocs; Brilliant Crios	Celtra Duo	Red wine, coffee, coke, energy drink, and distilled water	30d immersion; Renewal: 1d; T: 37 °C	Baseline, 1d, 7d, 30d after immersion	Spectrophotometer (Vita Easy Shade Advance, Germany)	n	/	∆E00 = 1.3 (p); ∆E00 = 2.25 (a)	After 30 d, all materials exposed to wine and coffee showed color change above the clinically acceptable value (ΔE00 = 2.25). Celtra Duo (Zirconia-reinforced lithium silicate) showed highest color stability
Aydin et al., 2021 [22]	Grandio Blocs; Brilliant Crios	Vita Enamic	Coffee	7d immersion; Renewal: 1d; T: 37 °C	Baseline, 1d, 7d after immersion	Spectrophotometer (Vita Easyshade V; VITA Zahnfabrik, Germany)	y	/	∆E00 = 0.8 (p); ∆E00 = 1.8 (a)	Brilliant Crios and Grandio Blocs unpolished specimen showed color change beyond clinical acceptability (>∆E00 = 1.8). All polished specimens showed perceptible color change but were clinically acceptable.
Barutçug et al., 2019 [14]	Lava Ultimate; Cerasmart	Vita Enamic	Red wine, coffee, distilled water	30d immersion; Renewal: 1d	Baseline, 1d, 30d	Spectrophotometer (VITA Easyshade Compact; VITA Zahnfabrik)	y	/	∆E00 = 2.25 (a)	After 1 month of immersion in coffee and red wine, a discoloration higher than the clinically acceptable threshold level (ΔE00 = 2.25) was observed for all tested CAD/CAM materials
Dalforno et al., 2022 [24]	Brava block	Vita Enamic; Vitablocks Mark II	Red wine	30 min immersions twice a day for 30 days; T: 37 °C	Baseline, 15d, 30d after immersion	Spectrophotometer (SP60, X-Rite, Grand Rapids, USA)	y	/	∆E00 = 0.8 (p); ∆E00 = 1.8 (a)	Brava Bloc and Vita Enamic showed significantly higher color change than Vita Mark II.
Eldwakhly et al., 2019 [15]	Lava Ultimate	IPS-e.max-CAD; Celtra Duo; Lava Plus; Vita Enamic	Coffee, coke, ginger, distilled water	28d immersion; Renewal: 1d; T: 37 °C	Baseline, 28d	Spectrophotometer (model RM200QC; X-Rite GmbH, Neu-Isenburg, Germany)	y	/	∆E = 1.2 (p); ∆E = 2.7 (a)	The color change was staining-solution- and material-dependent, with IPS-e.max-CAD showing the greatest color stability. Lava Plus stained with ginger and coffee showed a clinically unacceptable color change. The Lava Ultimate materials were most affected by the coffee and ginger solutions, whereas the Celtra Duo was affected by cola drinks.
Elsaka et al., 2022 [25]	Grandio Blocs; Lava Ultimate	/	Coffee, tea, coke, ginger, distilled water	7d immersion; Renewal: 2d; T: 37 °C	Baseline, 7d, after bleaching	Spectrophotometer (VITA Easyshade Advance 4.0, VITA Zahnfabrik, Bad Säckingen, Germany)	y	/	∆E00 = 1.8 (a)	Lava Ultimate revealed higher color changes than Grandio Blocs. Staining beverage solutions had a distinct influence on the optical properties of the tested CAD/CAM restorative materials.
Jalali et al., 2022 [26]	Mazic Duro	Vita Enamic; Vita Mark II	Carrot juice, coffee, distilled water	30d immersion; Renewal: 3d; T: 37 °C	Baseline, 30d	Spectrophotometer (X-Rite I1-Pro, X-Rite, Grand Rapids, USA)	y	/	∆E ^4^ = 3.3 (a)	The color change of all ceramic specimens was within the clinically acceptable range, except for the glazed Mazic Duro ceramic specimens immersed in carrot juice. However, the color difference of Vita Enamic and Mazic Duro was higher than that of feldspathic porcelain.
Kang et al., 2020 [27]	Cerasmart 200; Cerasmart 300; KZR-CAD HR; KZR-CAD HR3; Estelite Block; Estelite P Block; Katana Avencia Block; Katana Avencia P Block; Mazic Duro; Duro Ace	/	10% ethanol, simulated red wine, deionized water	12w immersion; Renewal: 1w; T: 37 °C	Baseline, 12w	Spectrophotometer (CiXX0, X-rite, USA)	y	/	∆E = 3.0	The tested reinforced hybrid blocks (except Duro Ace and Estelite P Block) showed lower color stability than their regular hybrid block counterparts. Estelite Block/Estelite P Block and Mazic Duro/Duro Ace showed better stain resistance than the others investigated materials
Koçak et al., 2021 [13]	Cerasmart	Vita Enamic; Cerec Blocs (Feldspatic); IPS-e.max-CAD	Tea, coffee, red wine, water	1, 7, and 30 days	Baseline, 1d, 7d, 30d	Spectrophotometer (SpectroShade Micro II; MHT Corp)	y	/	∆E = 2.65 (a); ∆E00(1:1:1) = 1.76 (a); ∆E00(2:1:1) = 1.78 (a)	Cerasmart (and Vita Enamic) CAD-CAM materials showed clinically unacceptable color change. LiDiSi showed highest color stability.
Lawson and Burgess, 2022 [37]	Lava Ultimate; Paradigm MZ 100	Vita Enamic; Paradigm C; IPS-e.max-CAD	Cranberry juice, tea, coffee	12d immersion; T: 37 °C	Baseline, 12d	Spectrophotometer (CM-700d; Konica Minolta, Ramsey, NJ, USA)	y	/	∆E00 = 1.25 (p); ∆E00= 2.23 (a)	The hybrid materials showed less stain resistance than IPS e.max CAD. When polished, however, all materials showed clinically acceptable color change following 1 year of artificial staining.
Quek et al., 2018 [12]	Lava Ultimate; Shofu HC block	Filtek Z350XT; Shofu Ceramage; Vita Enamic	Cola, tea, coffee, red wine, distilled water	7d immersion; renewal: 2d; T: 37 °C	baseline, 7d	Spectrophotometer (Konica Minolta CM-2600D, Tokyo, Japan)	y	/	∆E = 3.3 (a)	CAD/CAM composites (Shofu HC Block; Lava Ultimate; Vita Enamic) showed higher clinical stability in red wine when compared to direct and indirect composites. Nevertheless, almost all materials evaluated suffered a clinically unacceptable change (∆E > 3.3) when exposed to red wine, tea, and coffee.
Sarıkaya et al., 2018 [16]	Lava Ultimate	Vita Enamic	Cola, tea, coffee, distilled water	2d immersion; T: 37 °C	baseline, 2d	Spectrophotometer (Vita Easy Shade Advance, Vita Zahnfab- rik, Germany)	y	/	∆E = 2.7 (a)	Both of the Lava Ultimate specimens stored in coffee and tea had higher ΔE* values than the Lava Ultimate specimens stored in the cola.
Schürmann and Olms, 2018 [18]	Lava Ultimate	Vita Enamic	Coffee, cola, red wine, distilled water	14d immersion; renewal: 2d; T: room T	baseline, 14d	Spectrophotometer Vita Easyshade 4.0	n	/	∆E = 2.7 (a); ∆E = 3.7 (a)	Investigated materials (Lava Ultimate; Vita Enamic) are particularly vulnerable to coffee and red wine with regard to shade stability.
Seyidaliyeva et al., 2020 [38]	Grandio Blocs	Vita Enamic; IPS-e.max-CAD	Red wine, curry, black tea, cola, water	4 week immersion; renewal: 2d; T: 37 °C	baseline, after termocycling, after 2w and 4w storage in staining solution	Spectroradiameter (SR, SpectraScan PR-650, MS- 75 lens, Photo Research Inc. Chatsworth, California)	y	/	∆E00 = 0.8 (p); ∆E00 = 1.8 (a)	Grandio Blocs shows the highest color change, followed by Vita Enamic and IPS e.max CAD. By polishing, discolorations of the above-mentioned materials could be considerably reduced.
Schürmann and Olms, 2018 [17]	Lava Ultimate	Vita Enamic; Vita Blocs Mark II; CAD-Temp	Coffee, cola, red wine, distilled water	14d immersion; renewal: 3.5d; T: room T	baseline, 14d	Spectrophotometer VITA Easyshade Advance 4.0 (VITA Zahnfabrik, Bad Säckingen, Germany)	n	/	∆E = 2.7 (a)	Lava Ultimate showed higher color change than Vita Enamic. After 14 days of immersion, shade differences which exceeded the clinical acceptance threshold of ΔE = 2.7 were shown by CAD-Temp in Coca-Cola, by Mark II in coffee, Coca-Cola and red wine, and by Vita Enamic and Lava Ultimate in coffee and red wine.
Silva et al., 2021 [28]	Lava Ultimate	Filtek Z350XT; IPS e.max Press	Coffee, distilled water	3 h/day for 30 days; T: 37 °C	baseline, 30d	Spectrophotometer (PCB 6807, BYK Gardner)	y	/	∆E00 = 0.8 (p); ∆E00 = 1.8 (a)	The Lava Ultimate showed intermediate staining and roughness compared to the Filtek Z350 and the IPS emax Press, the latter showing the best optical and physical properties.
Stamenkovic et al., 2021 [19]	Cerasmart; Lava Ultimate; Shofu HC	IPS e.max CAD; Vita Enamic; Vita Suprinity	Coffee, red wine, accelerated artificial aging	2.5 (T1) and 5 (T2) days; renewal: 1d; T: 37 °C	baseline, 5d	Spectrophotometer Ci7600 (X-Rite)	y	/	∆E00 = 0.8 (p); ∆E00 = 1.8 (a)	Coffee caused the greatest color changes for T0-T2 interval. Staining-dependent color differences increased with increased exposure, except for IPS e.max and Vita Suprinity. For artificial aging, color change appeared to be dependent on material.

**Table 4:** Color change of CAD/CAM composite blocks investigated by the papers included in the current review. (1: ∆E00 = color change obtained with CIEDE2000 formula; 2: ∆E= color change value obtained with CIELAB formula).

First Author, Year	Clinical Threshold (Perceptible = p; Acceptable = a)	Lava Ultimate	Paradigm MZ 100	Brilliant Crios	Crystal Ultra	Brava Block	Cerasmart	Cerasmart 300	Katana Avencia Block	Katana Avencia P Block	Shofu HC Block	Estelite Block	Estelite P Block	Duro Ace	Mazic Duro	Grandio Blocs	KZR-CAD HR2	KZR-CAD HR3	*p*
Acar et al., 2016 [35]	∆E00 ^1^ = 1.28 (p); ∆E00 = 2.24 (a)	2.24 < ∆E00 < 6 (data extracted from graph)	\	\	\	\	\	\	\	\	\	\	\	\	\	\	\	\	<0.05
Al Amri et al., 2021 [36]	∆E00 = 0.8 (p); ∆E00 = 1.8 (a)	T1: 2.2 < ∆E00 < 3; T2: 1.2 < ∆E00 < 1.7 (data extracted from graph)	\	\	T1: 1.2 < ∆E00 < 2; T2: 0.7 < ∆E00 < 1.2 (data extracted from graph)	\	T1: 1.7 < ∆E00 < 2.6; T2: 0.8 < ∆E00 < 1.5 (data extracted from graph)	\	\	\	\	\	\	\	\	\	\	\	<0.05
Aydin et al., 2020 [23]	∆E00 = 1.3 (p); ∆E00 = 2.25 (a)	\	\	Wine: ∆E00 = 8.69 ± 0.93; Coffee: ∆E00 = 2.43 ± 0.52; Coke: ∆E00 = 0.77 ± 0.21; Energy drink: ∆E00 = 0.60 ± 0.17	\	\	Wine: ∆E00 = 6.46 ± 1.10; Coffee: ∆E00 = 2.31 ± 0.29; Coke: ∆E00 = 0.51 ± 0.22; Energy drink: ∆E00 = 0.59 ± 0.08	\	\	\	Wine: ∆E00 = 6.63 ± 0.88; Coffee: ∆E00 = 2.38 ± 0.22; Coke: ∆E00 = 0.52 ± 0.10; Energy drink: ∆E00 = 0.37 ± 0.06	\	\	\	\	Wine: ∆E00 = 8.69 ± 0.93; Coffee: ∆E00 = 2.43 ± 0.52; Coke: ∆E00 = 0.60 ± 0.21; Energy drink: ∆E00 = 0.60 ± 0.17	\	\	<0.05
Aydin et al., 2021 [22]	∆E00 = 0.8 (p); ∆E00 = 1.8 (a)	\	\	One step: ∆E00 = 1.0 ± 0.2; One step + paste: ∆E00 = 0.9 ± 0.1; Two step: ∆E00 = 1.0 ± 0.1; Two step + paste: ∆E00 = 0.9 ± 0.1; Multi step: ∆E00 = 1.7 ± 0.1; Multi step + paste: ∆E00 = 1.4 ± 0.1	\	\	.	\	\	\	\	\	\	\	\	One step: ∆E00 = 1.3 ± 0.2; One step + paste: ∆E00 = 1.2 ± 0.2; Two step: ∆E00 = 1.3 ± 0.2; Two step + paste: ∆E00 = 1.2 ± 0.2; Multi step: ∆E00 = 1.6 ± 0.1; Multi step + paste: ∆E00 = 1.3 ± 0.2	\	\	<0.05
Barutçug et al., 2019 [14]	∆E00 = 2.25 (a)	Wine: ∆E00 = 3.5 ± 0.3; Coffee: ∆E00 = 3.2 ± 0.5	\	\	\	\	Wine: ∆E00 = 2.7 ± 0.7; Coffee: ∆E00 = 3.1 ± 1.1	\	\	\	\	\	\	\	\	\	\	\	<0.05
Dalforno et al., 2022 [24]	∆E00 = 0.8 (p); ∆E00 = 1.8 (a)	\	\	\	\	∆E00 = 5.49 ± 0.73	\	\	\	\	\	\	\	\	\	\	\	\	<0.05
Eldwakhly et al., 2019 [15]	∆E ^2^ = 1.2 (p); ∆E = 2.7 (a)	∆E = 1.59 ± 0.66	\	\	\	\	\	\	\	\	\	\	\	\	\	\	\	\	<0.05
Elsaka et al., 2022 [25]	∆E00 = 1.8 (a)	Tea: ∆E00 = 2.8 ± 0.2; Coffee: ∆E00 = 3.1 ± 0.2; Coke: ∆E00 = 2.5 ± 0.2; Ginger: ∆E00 = 2.7 ± 0.2 (data extracted from graph)	\	\	\	\	\	\	\	\	\	\	\	\	\	Tea: ∆E00 = 2.4 ± 0.2; Coffee: ∆E00 = 2.6 ± 0.2; Coke: ∆E00 = 2.1 ± 0.2; Ginger: ∆E00 = 2.3 ± 0.2 (data extracted from graph)	\	\	<0.05
Jalali et al., 2022 [26]	∆E = 3.3 (a)	\	\	\	\	\	\	\	\	\	\	\	\	\	Polished: (carrot juice: ∆E = 1.63 ± 0.76; coffee: ∆E = 1.01 ± 0.75); glazed: (carrot juice: ∆E = 3.46 ± 2.66; coffee: ∆E = 3.05 ± 2.28)	\	\	\	<0.05
Kang et al., 2020 [27]	∆E = 3.0	\	\	\	\	\	10% ethanol: ∆E = 0.93 ± 0.39; Wine: ∆E = 7.16 ± 1.15	10% ethanol: ∆E = 1.52 ± 0.49; Wine: ∆E = 7.16 ± 1.15	10% ethanol: ∆E = 0.58 ± 0.10; Wine: ∆E = 2.07 ± 0.25	10% ethanol: ∆E = 3.51 ± 0.06; Wine: ∆E = 8.50 ± 0.81	\	10% ethanol: ∆E = 1.45 ± 0.23; Wine: ∆E = 4.52 ± 0.53	10% ethanol: ∆E = 0.93 ± 0.19; Wine: ∆E = 4.45 ± 0.27	10% ethanol: ∆E = 1.84 ± 0.38; Wine: ∆E = 3.51 ± 0.56	10% ethanol: ∆E = 1.38 ± 0.01; Wine: ∆E = 3.95 ± 0.29	\	10% ethanol: ∆E = 1.13 ± 0.38; Wine: ∆E = 5.58 ± 0.90	10% ethanol: ∆E = 1.51 ± 0.75; Wine: ∆E = 9.59 ± 1.71	<0.05
Koçak et al., 2021 [13]	∆E00(1:1:1) = 1.76 (a)	\	\	\	\	\	Wine: ∆E00 = 18 ± 1; Coffee: ∆E00 = 6 ± 1; Tea: ∆E00 = 3 ± 1 (data extracted from graph)	\	\	\	\	\	\	\	\	\	\	\	<0.05
Lawson and Burgess, 2022 [37]	∆E00 = 1.25 (p); ∆E00 = 2.23 (a)	Polished: ∆E00 = 1.51 ± 0.51; Un-polished: ∆E00 = 2.76 ± 1.19	Polished: ∆E00 = 0.58 ± 0.34; Un-polished: ∆E00 = 1.23 ± 0.25	\	\	\	\	\	\	\	\	\	\	\	\	\	\	\	<0.05
Quek et al., 2018 [12]	∆E = 3.3 (a)	Tea: ∆E = 3.16 ± 0.45; Coffee: ∆E = 4.01 ± 0.48; Coke: ∆E = 0.56 ± 0.19; wine: ∆E = 6.30 ± 1.47	\	\	\	\	\	\	\	\	Tea: ∆E = 5.42 ± 0.51; Coffee: ∆E = 4.08 ± 0.38; Coke: ∆E = 0.73 ± 0.18; wine: ∆E = 5.55 ± 0.59	\	\	\	\	\	\	\	<0.05
Sarıkaya et al., 2018 [16]	∆E = 2.7 (a)	polishing (Tea: ∆E = 2.69 ± 0.59; Coffee: ∆E = 3.35 ± 0.40; Coke: ∆E = 1.89 ± 0.24); Sof-lex (Tea: ∆E = 3.43 ± 0.27; Coffee: ∆E = 3.84 ± 0.85; Coke: ∆E = 2.75 ± 0.28); Shofu (Tea: ∆E = 3.55 ± 0.36; Coffee: ∆E = 3.87 ± 0.46; Coke: ∆E = 2.81 ± 0.35)	\	\	\	\	\	\	\	\	\	\	\	\	\	\	\	\	<0.05
Schürmann and Olms. 2018 [18]	∆E = 2.7 (a); ∆E = 3.7 (a)	Coffee 1.5 < ∆E < 2; Coffee + simulated chewing 2.7 < ∆E < 4.3 (data extracted from graph)	\	\	\	\	\	\	\	\	\	\	\	\	\	\	\	\	<0.05
Seyidaliyeva et al., 2020 [38]	∆E00 = 0.8 (p); ∆E00 = 1.8 (a)	\	\	\	\	\	\	\	\	\	\	\	\	\	\	∆E00 = 5.0 ± 4.5	\	\	<0.05
Schürmann and Olms, 2018 [17]	∆E = 2.7 (a)	Wine: ∆E = 8.61 ± 0.30; Coffee: ∆E = 6.08 ± 0.76; Coke: ∆E = 1.32 ± 0.14	\	\	\	\	\	\	\	\	\	\	\	\	\	\	\	\	<0.05
Silva et al., 2021 [28]	∆E00 = 0.8 (p); ∆E00 = 1.8 (a)	∆E00 = 2.5 ± 0.5	\	\	\	\	\	\	\	\	\	\	\	\	\	\	\	\	<0.05
Stamenkovic et al., 2021 [19]	∆E00 = 0.8 (p); ∆E00 = 1.8 (a)	Coffee: ∆E00 = 6.5 ± 1.0; Wine: ∆E = 2.8 ± 0.4	\	\	\	\	Coffee: ∆E00 = 3.1 ± 0.5; Wine: ∆E = 1.6 ± 0.2	\	\	\	Coffee: ∆E00 = 5.0 ± 0.3; Wine: ∆E = 2.8 ± 0.3	\	\	\	\	\	\	\	<0.05

**Table 5:** Risk of Bias.

	Clearly Stated Aims/Objectives	Detailed Explanation of Sample Size Calculation	Detailed Explanation of Sampling Technique	Details of Comparison Group	Detailed Explanation of Methodology	Operator Details	Randomization	Method of Measurement of Outcome	Outcome Assessor Details	Blinding	Statistical Analysis	Presentation of Results	Total Score	Final Score %	Risk of Bias
Acar et al. [36]	2	1	0	0	2	0	0	2	0	0	2	2	11	45.83	high
Al Amri et al. [37]	2	2	1	2	2	0	2	2	0	2	2	2	19	79.16	low
Aydin et al. [22]	2	0	1	0	1	0	0	2	0	0	2	2	10	41.66	high
Aydin et al. [23]	2	1	0	0	1	0	0	2	0	0	2	2	10	41.66	high
Barutcugil et al. [14]	2	2	1	1	2	0	0	1	0	0	2	2	13	54.16	medium
Dalforno et al. [24]	2	2	1	0	2	0	0	2	0	0	2	2	13	54.16	medium
Eldwakhly et al. [15]	2	1	2	0	2	0	2	2	0	0	2	2	15	62.5	medium
Elsaka et al. [25]	2	2	2	1	2	0	0	2	0	0	2	2	15	62.5	medium
Jalali et al. [26]	2	2	1	0	2	1	0	2	0	0	2	2	14	58.33	medium
Kang et al. [27]	2	1	2	0	2	0	2	2	0	0	2	2	15	62.5	medium
Kocak et al. [13]	2	1	1	1	2	0	0	2	0	0	2	2	13	54.16	medium
Lawson and Burgess et al. [38]	2	2	2	0	2	0	0	2	0	0	2	2	14	58.33	medium
Quek et al. [12]	2	2	2	0	2	0	0	2	0	0	2	2	14	58.33	medium
Sarikaya et al. [16]	2	2	2	1	2	0	0	2	0	0	2	2	15	62.5	medium
Schurmann and Olms et al. [18]	2	0	1	1	2	0	0	2	0	0	2	2	12	50	medium
Schurmann and Olms et al. [17]	2	1	1	1	2	1	0	2	0	0	2	2	14	58.33	medium
Seydaliyeva et al. [39]	2	2	2	0	2	0	1	2	0	0	2	2	15	62.5	medium
Silva et al. [28]	2	2	2	2	2	0	2	2	0	0	2	2	18	75	low
Stamenkovic et al. [19]	2	2	1	0	2	1	2	2	0	0	2	2	16	66.66	medium

## Data Availability

Not applicable.
